# Applying high‐throughput sequencing to identify and evaluate foetal chromosomal deletion and duplication

**DOI:** 10.1111/jcmm.15593

**Published:** 2020-07-15

**Authors:** Yueli Wu, Linlin Zhang, Hong Lv, Ying Li, Chongyang Zhu, Weifang Tian, Ling Zhao

**Affiliations:** ^1^ Prenatal Diagnosis Center of Henan Women and Children Hospital and Institute the Third Affiliated Hospital of Zhengzhou University Zhengzhou China; ^2^ Clinical Laboratory of Henan Women and Children Hospital and Institute the Third Affiliated Hospital of Zhengzhou University Zhengzhou China

**Keywords:** cell‐free foetal DNA, chromosomal abnormalities, chromosomal deletions/duplications, high‐throughput sequencing, non‐invasive prenatal testing

## Abstract

The present study aimed to estimate the clinical performance of non‐invasive prenatal testing (NIPT) based on high‐throughput sequencing method for the detection of foetal chromosomal deletions and duplications. A total of 6348 pregnant women receiving NIPT using high‐throughput sequencing method were included in our study. They all conceived naturally, without twins, triplets or multiple births. Individuals showing abnormalities in NIPT received invasive ultrasound‐guided amniocentesis for chromosomal karyotype and microarray analysis at 18‐24 weeks of pregnancy. Detection results of foetal chromosomal deletions and duplications were compared between high‐throughput sequencing method and chromosomal karyotype and microarray analysis. Thirty‐eight individuals were identified to show 51 chromosomal deletions/duplications via high‐throughput sequencing method. In subsequent chromosomal karyotype and microarray analysis, 34 subchromosomal deletions/duplications were identified in 26 pregnant women. The observed deletions and duplications ranged from 1.05 to 17.98 Mb. Detection accuracy for these deletions and duplications was 66.7%. Twenty‐one deletions and duplications were found to be correlated with the known abnormalities. NIPT based on high‐throughput sequencing technique is able to identify foetal chromosomal deletions and duplications, but its sensitivity and specificity were not explored. Further progress should be made to reduce false‐positive results.

## 1 INTRODUCTION

Deletions and duplications can result in abnormalities in chromosomal structure and function, thus leading to a wide range of congenital anomalies, such as Cri du chat syndrome, DiGeorge syndrome, etc.^1^, ^2^, ^3^ Approximate 1 in 150 live births presents chromosomal abnormalities.^4^ Although the mortality is not extremely high, survivors have severe disabilities.^5^ Even worse, there are no specific treatments for such chromosomal disorders until now. Chromosomal deletions and duplications can be prenatally detected using foetal DNA samples, which may be the best way to prevent chromosomal abnormalities for newborns.^6^ Conventional prenatal testing techniques include karyotyping, comparative genomic hybridization (CGH), hybridization and array‐based technologies.^7^ These conventional screening methods require foetal DNA samples through invasive approaches, like amniocentesis, which may increase the risk of miscarriage and infection.^8^ Moreover, conventional testing techniques could only detect deleted and duplicated fragments of more than 10 Mb, and abnormalities with microdeletion and microduplication may be undetectable.^9^ Therefore, non‐invasive prenatal genetic screening methods with high accuracy are in urgent need.

In 1997, Lo et al reported the presence of cell‐free foetal DNA (cffDNA) in maternal plasma that allows the application of non‐invasive prenatal testing (NIPT) in clinical practice.^10^ cffDNA in maternal plasma mainly derives from placenta, especially from the outer cytotrophoblastic layer.^11^ cffDNA shows linear correlation with chromosomal abnormalities in foetuses and is considered as the optimal proxy in NIPT.^12^ NIPT based on high‐throughput sequencing technique can effectively detect large‐scale genetic mutations in a short time, with high accuracy.^13^ Compared to conventional prenatal testing, high‐throughput NIPT has multiple advantages. First, it causes no risk of pregnancy loss thanks to its non‐invasive procedures.^14^ Second, it has been reported that the detection rate of high‐throughput sequencing for trisomy 21, trisomy 18 and trisomy 13 may be up to 79%.^15^ Third, the technique is suitable for varied gestational ages, even after 23 weeks of pregnancy. In addition, the operational process is simple and automated. However, the technique is not suitable in the detection for multiple births. Moreover, its detection rate for deletions and duplications less than 10 Mb is unsatisfactory. The performance of NIPT using high‐throughput sequencing technique for chromosomal deletions and duplications remained controversial.

In this study, we estimated the performance of NIPT based on high‐throughput sequencing for foetal deletions and duplications.

## 2 MATERIALS AND METHODS

### 2.1 Study subjects

A total of 6348 eligible pregnant women were retrospectively recruited in the current study from May 2015 to January 2019. The all conceived naturally, without twins, triplets or multiple births. The included pregnant women received NIPT which was performed via high‐throughput sequencing method, regardless of whether they experienced any Down syndrome examinations. Moreover, the age of the eligible subjects was over 18 years, with a pregnancy of more than 12 weeks. The pretest ultrasound scan was performed for each subject to confirm the number of foetuses and gestational age. In addition, women who had a foetus with major structural abnormalities were excluded from this study. Written informed consent was signed by each woman before inclusion. The current investigation was approved by the Ethics Committee of the Third Affiliated Hospital of Zhengzhou University.

### 2.2 Blood sample and DNA extraction

Five millilitres of peripheral blood from each pregnant woman was collected into a cell‐free DNA tube (Streck, Omaha, NE, USA). Then, cell‐free plasma was isolated from the obtained blood samples via a two‐step centrifugation method within 4 hours after collection. In brief, blood samples were first centrifuged at 1600 *g* for 10 minutes at 4°C, and then supernatant was transferred into a new tube and centrifuged for additional 10 minutes at 1600 *g* under 4°C. Final plasma supernatant was transferred to a cell‐free DNA tube and then stored at −20°C for DNA extraction. Each plasma sample was thawed only once.

Cell‐free DNA was extracted from plasma specimens using a Dynabeads^®^ Viral NA DNA purification kit (Dynal, Grand Island, NY, USA), and experiment procedures were performed based on the instruction of manufacturer. DNA samples were stored at −80°C.

### 2.3 DNA library construction

Firstly, cell‐free DNA samples were quantified by Qubit 3.0 fluorometer (Invitrogen, Life Technologies, Carlsbad, CA, USA). No less than 10 ng DNA sample was collected from each woman, and DNA concentration was over 1.7 ng/mL. Qualified DNA samples were adopted for PCR amplification, and reaction procedures were as follows: at 99°C for 2 minutes, and 22 cycles of 99°C for 15 seconds and 60°C for 4 minutes. After primer digestion, amplification products were ligated with adaptors and purified by Agecoure AMPure SPRI beads (Beckmancoulter, Brea, CA, USA). Subsequently, the library was amplified in a volume of 52 μL solution containing 50 μL PCR amplification mixture and 2 μL primers. Then, the library was purified by magnetic beads. DNA concentration of library was estimated by Qubit 3.0 fluorometer (Invitrogen, Life Technologies).

### 2.4 Sequencing template preparation and enrichment

Sequencing templates were prepared and enriched according to the standard procedures recommended by Life Technology Company. The template was prepared through emulsion PCR, which was performed using Ion temple preparation kit (Life Technologies). The reaction was carried out in a volume of 1 mL mixture including 582 μL nuclease‐free water, 200 μL 5× PCR reagent mix, 100 μL 10× PCR enzyme mix, 100 μL Ion Sphere particles (ISPs) and 18 μL diluted library template. The mixture was shaken and centrifuged, and Ultra‐Turrax tube drive (Life Technologies) was adopted for emulsion. Then, the mixed emulsion was transferred to 96‐well plate and amplified on an ABI 2720 thermocycler (Life Technologies).

After amplification, ISP was recovered using Ion Xpress template kit (Life Technologies) following the instruction of manufacturer. Qubit 3.0 fluorometer and Ion Sphere quality control kit were applied for particle quantification. The optimal positive ISPs for enrichment were 4%‐50%. Finally, ISP enrichment was performed using Ion Xpress template kit, Ion sequencing kit and DynaBeads MyOne streptavidin C1 beads (Life Technologies), and experiments were carried out according to the guidance of manufacturer.

### 2.5 Ion torrent proton sequencing

The prepared sequencing template was annealed on PCR amplification thermocycler, and the parameters were set as follows: 95°C for 2 minutes, 37°C for 2 minutes and 25°C for storage. The annealed template was loaded and run with 200‐bp single‐end run configuration based on the manufacturer's instruction (Ion sequencing kit user guide, version 2.0).

### 2.6 Bioinformatics analysis

Sequencing results were collected and analysed using VariantCaller software (Life Technologies). In comparison with human genome reference sequence (GRCh37 Sequences), chromosomal deletion and duplication were analysed. Reference genome was divided into 300 000 sliding windows which contained the same number of reads, and relative number of reads was defined as the ratio of the number of reads in an equal window to the average number of reads. The least square method was adopted to analyse linear relationship between GC content and relative number of reads. The types of foetal chromosome abnormality were predicted through dynamic threshold method and quadratic element segmentation algorithm.

### 2.7 Chromosome karyotype analysis and microarray analysis

Individuals who showed abnormalities in NIPT received ultrasound‐guided amniocentesis at 18‐24 weeks of pregnancy after informed consent was signed by pregnant women and their families. Amniotic fluid samples were collected from the patients for conventional G‐banded cytogenetic assays and microarray analysis. Amniocytes were isolated and cultured using BIO‐AMF™‐2 medium (Biological Industries, Kibbutz Beit‐Haemek, Israel) and Chang Medium^®^ D (Irvine Scientific, Santa Ana, CA, USA) at 37°C with 5% CO_2_ for 6‐7 days. The cells at metakinesis were harvested to prepare slides according to the statements in published article.^16^ Then, G‐band staining was performed according to the Internal System for Human Cytogenomic Nomenclature 2016.

In addition, microarray analysis was also performed for the patients. In brief, genomic DNA was extracted from amniotic fluids using Genomic DNA Extraction kit (QIAamp DNA Blood Mini kit; Qiagen GmBH, Hilden, Germany). DNA samples were digested, ligated and amplified via PCR method. Then, obtained products were processed according to standard procedures of Affymetrix CytoScan 750K Array analysis. The results were processed in Affymetrix GeneChip Command Console software (version 4.0) and Chromosome analysis software (Chromosome Analysis Suite version 2.1) (Affymetrix; Thermo Fisher Scientific, Inc).

## 3 RESULTS

### 3.1 Baseline characteristics of the study population

A total of 6348 pregnant women undergoing NIPT using high‐throughput sequencing method were included in our study. All of the eligible cases were Chinese, and 94.01% of them were Han population. The average age of the included cases was 32.65 ± 8.16 years, with an age range of 21‐48 years. 50.63% of subjects were pregnant for the first time, when 36.97% of individuals were for the second time. The median gestational age at NIPT was 14.23 weeks, and 54.44% of the participants received NIPT at 12‐13 weeks of gestation. Fifty‐six individuals (0.88%) had the history of trisomy 21 pregnancy, 46 cases (0.72%) had been affected by trisomy 18 at pregnancy, and 32 (0.5%) cases had previously underwent trisomy 13 pregnancies. Family history of trisomy 21 was observed in 72 (1.13%) cases. Approximate 75.8% of cases received screening test before NIPT. Meanwhile, 956 (19.87%) cases were confirmed at high risk, according to prior screening test, low risk was proposed for 3179 (66.06%) cases, while 577 (11.99%) individuals had no available data at the time of NIPT. Detailed information of the included subjects was summarized in Table 1.

**TABLE 1:** Basic characteristics of 6348 pregnant women undergoing NIPT based on high‐throughput sequencing method

Characteristics	n (%)
Ethnicity	
Chinese Han	5968 (94.01)
Minorities	380 (5.99)
Age (y)	
20‐24	1056 (16.64)
25‐29	2008 (31.63)
30‐34	2141 (33.73)
35‐39	821 (12.93)
40‐44	292 (4.6)
≥45	30 (0.47)
Mean age (y)	32.65 ± 8.16
Age range (y)	21‐48
Number of pregnancy	
One	3214 (50.63)
Two	2347 (36.97)
Three	592 (9.33)
More than three	195 (3.07)
Gestation at NIPT	
12 to 13+6 wk	3456 (54.44)
14 to 15+6 wk	2267 (35.71)
16 to 20+6 wk	501 (7.89)
≥21 wk	124 (1.95)
Previous trisomy 21 pregnancy	56 (0.88)
Previous trisomy 18 pregnancy	46 (0.72)
Previous trisomy 13 pregnancy	32 (0.5)
Family history of trisomy 21	72 (1.13)
Prior Down syndrome screening test	
None	1536 (24.2)
Combined first‐trimester NT + biochemistry	3671 (57.83)
First‐trimester NT (±other ultrasound markers) only	986 (15.53)
First‐trimester biochemistry only	60 (0.95)
Second‐trimester biochemistry only	60 (0.95)
Other tests, or more than one test	35 (0.55)
Result of prior screening tests	
High risk	956 (19.87)
Low risk	3179 (66.06)
Result not available at time of NIPT	577 (11.99)

Abbreviations: NIPT, non‐invasive prenatal testing.

### 3.2 Overall results of NIPT

**FIGURE 1:**
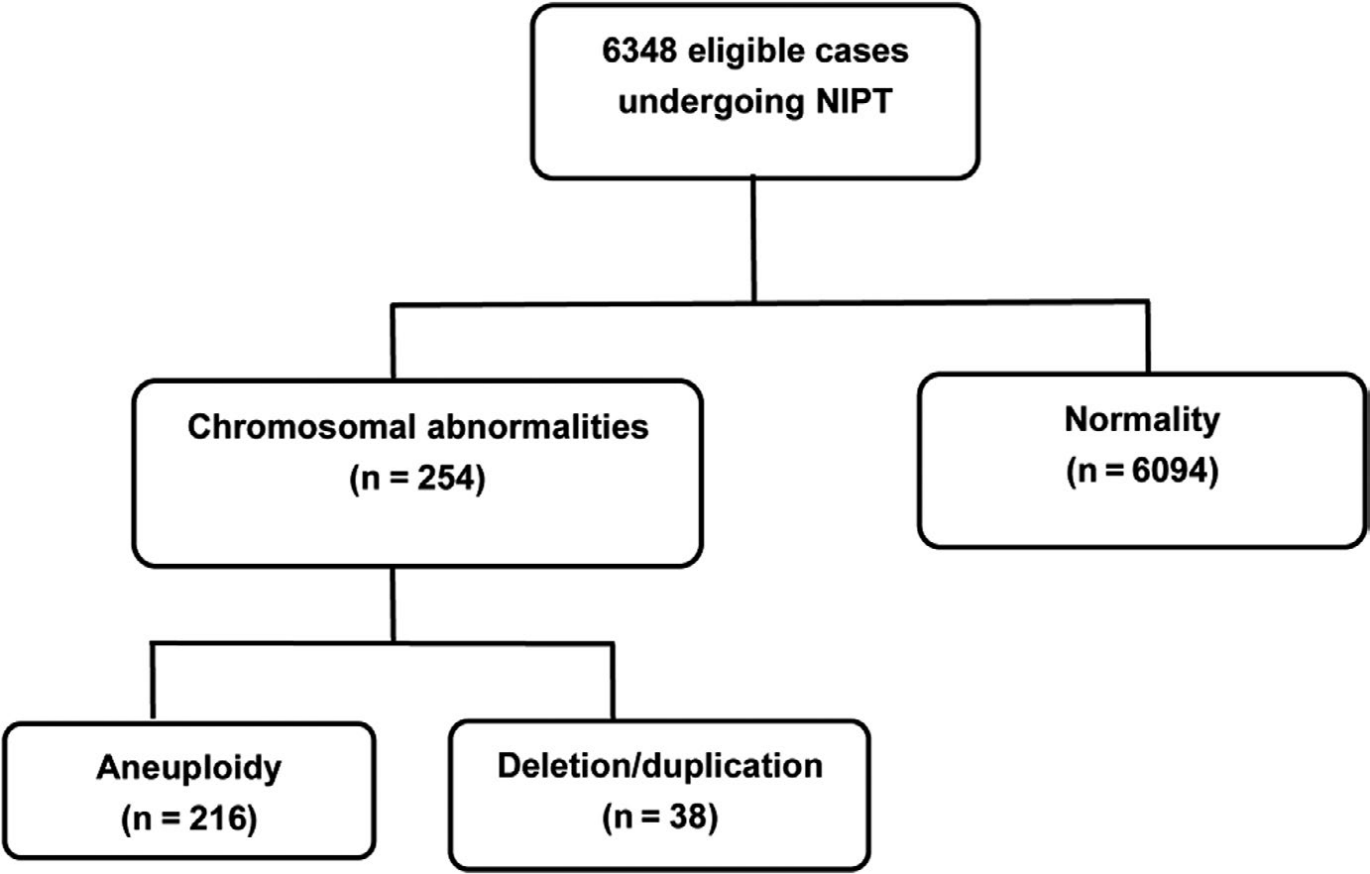
Diagram summarizing the non‐invasive prenatal testing results of the included pregnant women. Chromosomal abnormalities were observed in 254 individuals, accounting for 4.0%. Chromosomal deletion/duplication was observed in 38 cases, accounting for 0.59%

**FIGURE 2:**
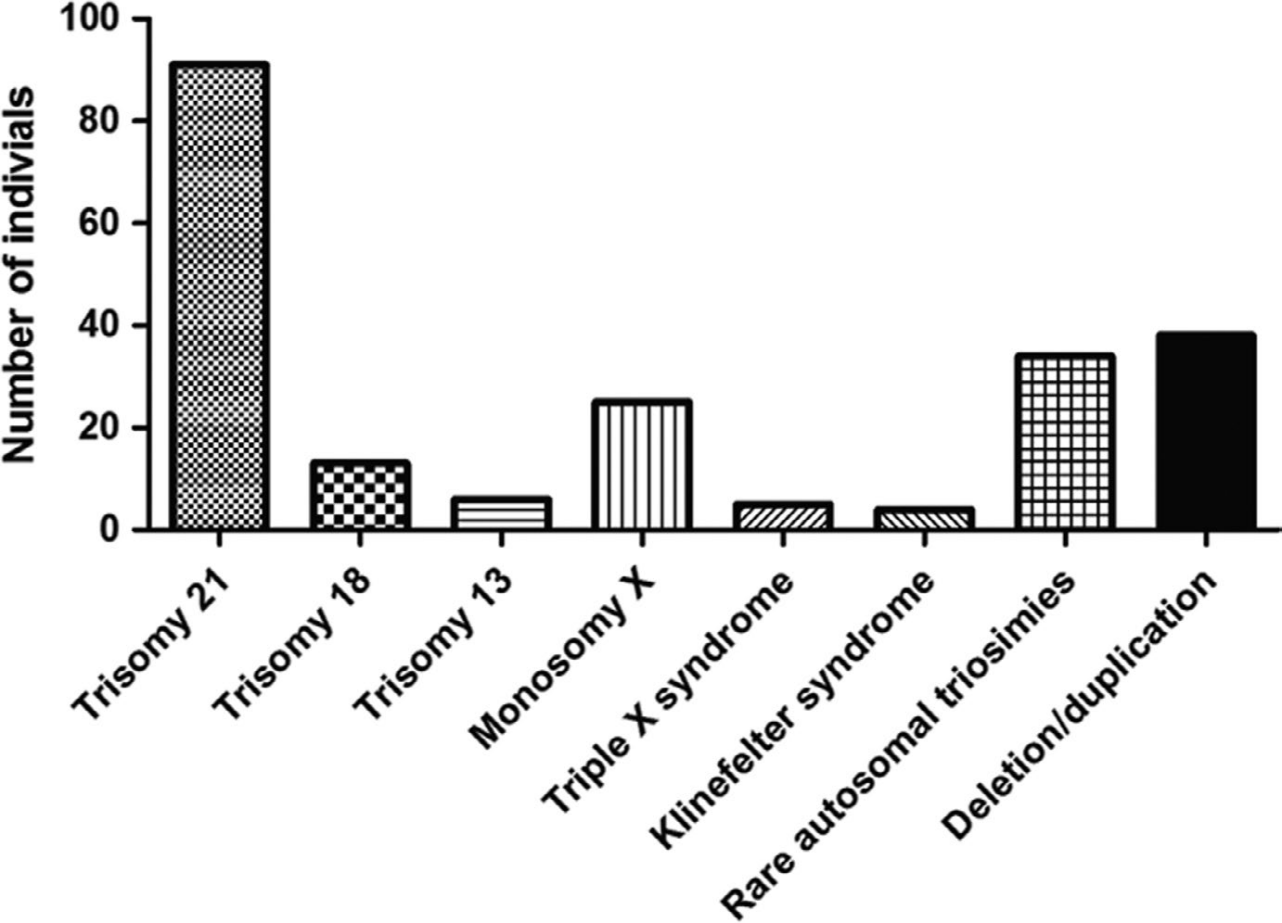
The types of chromosomal abnormalities in the study population

The results of NIPT based on high‐throughput sequencing method were available for all of the included patients. As displayed in Figure 1, 6094 (96.00%) patients were normal, while the rest 254 patients showed chromosomal abnormalities or suspected abnormalities, accounting for 4.0% of whole cases. Common aneuploidy was observed in 216 patients (3.40%), while chromosomal deletion/duplication was observed in 38 subjects, accounting for 0.59% of whole cases. Among those with chromosomal aneuploidy, 91 (1.43%) showed trisomy 21, 13 (0.2%) had trisomy 18, and 6 (0.09%) had trisomy 13. Monosomy X was observed in 25 cases (0.39%), triple X syndrome was observed in five individuals (0.08%), and four cases (0.06%) exhibited Klinefelter syndrome. Furthermore, 34 individuals showed rare autosomal triosimies, accounting for 0.53% of whole cases (Figure 2).

### 3.3 Detective performance of NIPT based on high‐throughput sequencing method for chromosomal deletions and duplications

In our study population, 38 individuals were identified to have 51 chromosomal deletions/duplications, according to NIPT with high‐throughput sequencing method, and all of them received subsequent invasive amniocentesis for chromosome karyotype and microarray analysis. Of them, 11 had more than one copy number variation (CNV). Thirty‐four subchromosomal deletions/duplications were identified in 26 pregnant women using the methods of chromosome karyotype and microarray analysis. Of them, seven had more than one CNV. The detection accuracy was 66.7% (34/51).

Comparison results between NIPT and amniocentesis detection were summarized in Table 2. Thirty‐four subchromosomal deletions and duplications were identified, and their sizes ranged from 1.05 to 17.98 Mb. No deletions/duplications less than 1 Mb were observed. Twenty‐one deletions and duplications could be annotated by the known abnormalities, accounting for 61.8% (21/34).

**TABLE 2:** The comparisons of NIPT and chromosome karyotype and microarray analysis for chromosomal deletions/duplications

Patient no.	Deletion/duplication	Chromosome	Size (Mb)	NIPT	Chromosome karyotype and microarray analysis	Syndrome annotation
1	Deletion	Chr1	4.07	832 398‐4 658 333	814 245‐4 882 747	Unknown
2	Deletion	Chr11	13.6	121 100 200‐136 623 102	121 302 918‐134 937 328	Systemic retardation, cardiac dysplasia, respiratory distress and other pathogenic mutations
3	Duplication	Chr5	2.62	24 632 136‐27 255 789	24 412 210‐27 452 145	Unknown
4	Deletion	Chr14	2.45	40 542 267‐43 246 317	40 908 224‐43 356 102	Unknown
5	Deletion	Chr16	1.25	14 756 175‐16 201 100	14 856 202‐16 107 118	Neurocognitive disorder Susceptibility locus
6	Duplication	Chr15	5.58	20 000 120‐27 365 100	20 527 768‐26 109 968	Prader‐Willi syndrome (type 2)
	Deletion	Chr1	1.05	145 430 500‐146 800 000	145 432 765‐146 478 256	Thrombocytopenia‐absent radius syndrome
	Deletion	Chr3	6.53	137 100 652‐143 901 100	137 241 653‐143 768 720	Unknown
7	Duplication	Chr13	6.00	19 400 000‐25 896 725	19 436 286‐25 437 825	Pathogenic mutations
8	Duplication	Chr9	12.99	310 100 000‐320 786 500	300 261 257‐313 256 123	Unknown
	Deletion	ChrX	2.59	31 512 608‐33 508 426	31 026 178‐33 612 667	Xp21 deletion syndrome
	Deletion	Chr3	6.00	137 456 548‐143 632 100	137 538 908‐143 542 510	Unknown
9	Deletion	Chr22	2.58	18 900 000‐21 514 100	18 919 900‐21 498 520	DiGeorge syndrome
10	Deletion	Chr5	1.40	250 892 900‐1 620 500	254 652‐1 658 566	Cri du Chat syndrome
11	Deletion	Chr15	4.88	21 410 200‐26 340 000	21 321 794‐26 205 055	Prader‐Willi/Angelman syndrome
12	Duplication	Chr13	3.58	24 521 100‐28 320 000	24 768 204‐28 346 964	Unclear clinical significance, benign mutation tendency
	Duplication	Chr16	15.46	94 807‐15 400 700	94 807‐15 556 797	Unknown
13	Deletion	Chr21	2.32	44 782 200‐46 912 100	44 565 059‐46 880 878	Unknown
	Deletion	Chr13	5.38	109 710 674‐115 232 100	109 723 745‐115 107 733	Mental retardation, special face, microcephaly, hypotonia, low birth weight and genital abnormalities
14	Duplication	Chr11	17.98	117 000 000‐135 100 500	117 124 000‐135 109 200	Jacobsen syndrome
15	Duplication	Chr17	4.24	16 426 210‐20 668 010	16 429 920‐20 667 174	Smith‐Magenis syndrome
16	Deletion	Chr16	4.86	46 200‐4 9827 900	46 271‐4 904 686	Rubinstein‐Taybi syndrome
17	Deletion	Chr6	2.06	168 756 200‐170 897 102	168 832 500‐170 896 037	Dysplasia of brain structure
	Duplication	Chr11	4.26	21 323 518‐25 581 711	21 323 518‐25 581 711	Unknown
	Deletion	Chr13	6.99	96 200 980‐103 410 000	96 437 228‐103 424 298	Unknown
18	Duplication	Chr17	1.41	72 360 200‐73 800 641	72 364 514‐73 777 326	Cardiovascular malformations
19	Deletion	ChrY	3.82	6 688 000‐10 510 512	6 688 691‐10 511 314	Unknown
20	Duplication	Chr2	14.71	161 423 256‐176 131 678	161 423 992‐176 132 164	Unknown
21	Deletion	Chr4	9.18	61 550‐8 237 698	61 552‐9 237 101	Wol‐Hirschhorn syndrome
22	Duplication	Chr12	11.23	45 000‐11 279 000	45 001‐11 278 012	Unknown
23	Deletion	Chr17	3.30	15 540 980‐18 845 920	15 549 649‐18 845 678	Potocki‐Lupski syndrome
24	Duplication	ChrY	10.34	12 560 980‐22 916 600	12 571 053‐22 916 805	Azoospermia factor region a
25	Deletion	Chr18	7.99	50 413 100‐58 403 927	50 413 206‐58 403 399	18q Deletion syndrome
26	Deletion	Chr10	10.85	183 300‐11 040 600	183 492‐11 035 280	Hypoparathyroidism, sensorineural deafness, and renal disease (HDRS)

Abbreviation: NIPT, non‐invasive prenatal testing

In addition, 17 abnormalities in 12 cases were misdiagnosed in NIPT, and the false‐positive rate was 33.3%. Detailed descriptions for false‐positive results were listed in Table 3. The size of these deletions/duplications ranged from 0.50 to 4.37 Mb, and the major of them were less than 1.5 Mb.

**TABLE 3:** The false‐positive results of NIPT

Patient no.	Deletion/duplication	Chromosome	Size (Mb)	NIPT results
1	Duplication	Chr19	0.54	327 273‐863 300
2	Deletion	Chr16	0.52	29 673 900‐30 197 412
3	Duplication	Chr22	0.91	22 069 900‐22 980 200
4	Deletion	Chr7	0.46	64 612 879‐65 148 399
	Deletion	Chr3	0.83	27 300‐853 200
5	Deletion	Chr1	1.17	736 537‐1 910 067
	Deletion	Chr1	1.20	54 987 800‐56 191 192
	Duplication	Chr4	1.10	15 700 256‐16 800 235
6	Duplication	Chr22	1.99	18 980 800‐20 970 900
7	Deletion	Chr6	1.08	16 920 770‐17 998 800
8	Duplication	Chr13	1.24	10 109 720‐11 348 912
	Deletion	Chr11	1.00	15 698 700‐16 700 941
9	Deletion	Chr18	0.57	52 690 900‐53 256 090
	Deletion	Chr5	0.73	2 096 900‐2 800 317
10	Duplication	ChrY	4.37	6 568 900‐10 876 200
11	Duplication	Chr2	0.50	89 015 800‐89 512 200
12	Deletion	Chr17	1.17	72 370 765‐73 545 200

NIPT, non‐invasive prenatal testing.

## 4 DISCUSSION

Foetal chromosomal deletions and duplications are major reasons for developmental delay and intellectual disability.^17^ Golden standards for the detection of chromosomal abnormalities are chromosome karyotype or microarray assay using amniocentesis, which obviously increase the risk of miscarriage and infection.^8^ With the discovery of cffDNA in pregnant women, NIPT using high‐throughput sequencing method is widely adopted for clinical detection of chromosomal deletions and duplications. The technique shows high diagnostic accuracy for trisomy 21, 18 and 13.^18^ NIPT based on cffDNA has been recommended as a highly accurate approach for pregnant women with high risk of foetal aneuploidy by the American College of Obstetricians and Gynaecologists and the Society for Maternal‐Fetal Medicine science 2011.^19^ However, there are no adequate clinical data to support the clinical application of NIPT for the detection of chromosomal deletions and duplications. The present study was designed to estimate diagnostic performance of NIPT using high‐throughput sequencing for foetal chromosomal deletions and duplications. Analysis results demonstrated that 66.7% of chromosomal deletions and duplications detected by NIPT using high‐throughput sequencing could be verified by chromosome karyotype and microarray analysis. The observed CNVs ranged from 1.05 to 17.98 Mb. For abnormalities less than 1.5 Mb, false‐positive rate was high. The size of deleted and duplicated CNVs was the major determinant of detective accuracy of high‐throughput sequencing method.

Non‐invasive prenatal testing based on high‐throughput sequencing is widely used for pregnant women with high risk of chromosomal abnormalities. However, for deletions and duplications less than 10 Mb, its accuracy is relatively low. Recently, a number of studies have been devoted to improve diagnostic accuracy of NIPT for chromosomal deletions and duplications. Jensen et al^20^ reported that when genomic coverage became fourfold, which was approximate 20‐fold over that of standard aneuploidy detection, deletion with 3 Mb could also be detected. The study carried out by Zhao et al^21^ demonstrated that high‐throughput sequencing method for the detection of microdeletion/microduplication (3‐40 Mb) might achieve 94.4% sensitivity and 99.4% specificity through improving statistical methods. Rampasek et al designed a probabilistic Hidden Markov model which combined the imbalance of allelic ratios at SNP positions, parental genotypes to phase nearby SNPs and coverage depth. In simulation experiments, about 40% of CNVs with 50‐400 kb could be detected under a foetal DNA concentration of 13%.^22^ Taken together, foetal DNA concentration, the size of deletion and duplication fragments, statistical methods and coverage depth are key factors for detection accuracy of high‐throughput sequencing for foetal chromosomal deletions and duplications.^23^, ^24^, ^25^

In the current study, NIPT based on high‐throughput sequencing technique was performed for 6348 eligible pregnant women. 4.0% of pregnant women showed chromosomal abnormalities or suspected abnormalities according to NIPT detection, while chromosomal deletions and duplications were observed in 38 patients. Invasive amniocentesis was performed for these patients. After chromosome karyotype and microarray analysis, 26 patients were identified to have 34 chromosomal deletions and duplications. The detection accuracy was 66.7%. The detected CNVs ranged from 1.05 to 17.98 Mb, and no CNVs less than 1 Mb were observed. Furthermore, among misdiagnosed CNVs by sequencing method, their size ranged from 0.50 to 4.37 Mb, and the majority of them were less than 1.5 Mb. NIPT based on high‐throughput sequencing technique showed lower accuracy for chromosomal microdeletions and microduplications, showing a high false‐positive rate. Several reasons might be responsible for such phenomenon. First, the size of deleted and duplicated CNVs was relatively small. Second, foetal DNA concentration might be not high enough. Third, the detected abnormalities might be maternal ones. In the study of Yin et al,^26^ 63.6% of false‐positive results were caused by deletions/duplications present in maternal DNA. In addition, sequencing depth and bioinformatics analysis methods required further optimization. The study carried out by Yin et al^26^ reported that with a sequencing depth up to 10 million reads, the sensitivity of sequencing method could achieve 94.5% among abnormalities more than 1 Mb.

It was worth noting that only 61.8% of deletions and duplications identified in our study were correlated with the known abnormalities. Many abnormalities might be normal inherited mutations, without clinical significance. With the developments of sequencing technique and the accumulation of clinical researches, expended databases may explain those unknown abnormalities. Thus, doctors should spend time on explaining relevant test results to patients. In addition, several limitation of NIPT based on high‐throughput sequencing technique should be stated. Firstly, high costs limit its wide application in clinical practice. Secondly, the performance of NIPT for twins remained unclear.^27^ Besides, limited by current genome function, a certain proportion of NIPT results were inconclusive.^28^ Additionally, in our study only individuals showing abnormalities in NIPT received chromosome karyotype analysis and microarray analysis, considering invasive procedures of amniocentesis and reported low negative rate of NIPT. Individuals who might be misdiagnosed in NIPT did not experience chromosome karyotype analysis, and false‐negative rate of NIPT was not calculated. Therefore, much more progress should be made to translate NIPT results in clinical practice.

In conclusion, NIPT based on high‐throughput sequencing technique is able to identify foetal chromosomal deletions and duplications. However, due to the relatively low foetal DNA concentration, small abnormal fragments and limited sequencing depth, its false‐positive rate may be high.

## CONFLICT OF INTEREST

The authors have no conflicts of interest to disclose.

## AUTHOR CONTRIBUTIONS

**Yueli Wu:** Conceptualization (equal); Data curation (equal). **Linlin Zhang:** Conceptualization (equal); Data curation (equal). **Hong Lv:** Formal analysis (equal); Funding acquisition (equal). **Ying Li:** Resources (equal); Writing‐review & editing (equal). **Chongyang Zhu:** Formal analysis (equal); Resources (equal); Writing‐review & editing (equal). **Weifang Tian:** Methodology (equal); Writing‐original draft (equal). **Ling Zhao:** Conceptualization (equal); Writing‐original draft (equal).

## Data Availability

All data generated or analysed during this study are included in this article.
